# AD|ARC (Administrative Data | Agricultural Research Collection): Characteristics of farm households participating in agri-environment schemes in Wales, UK.

**DOI:** 10.23889/ijpds.v9i5.2750

**Published:** 2024-09-18

**Authors:** Sian Morrison-Rees, Rachael Loftus, Laura Madden, Freya Pryce, Matthew Curds, Paul Caskie

**Affiliations:** 1 Swansea University; 2 Welsh Government; 3 Agri Food and Biosciences Institute

## Objectives

Describe the sociodemographic and farm business characteristics of farming households in Wales that have agri-environment contracts (voluntarily opting-in) without primary farm subsidies (voluntarily opting-out).
Investigate the sociodemographic and farm business characteristics of farming households in Wales by the number and type of farming subsidies received.

## Approach

We utilised the AD|ARC dataset comprising of Census 2011 variables, linked to agricultural and business variables for all farming households in Wales.

We analysed variables relating to the individual (e.g. age, gender), household (e.g. household size, structure), farm business (e.g. farm type, diversification activities) and rural payments data (subsidy type, amount paid).

We conducted comparative analyses between three groups of farming households: households engaged in agri-environmental schemes but not primary subsidies, households engaged with both subsidy types, and households claiming primary subsidies only.

## Results

We will present a detailed description of the sociodemographic and business characteristics of farming households by the number and type of subsidies claimed and engagement with agri-environmental schemes.

Using business, personal, and household characteristics, we will discuss different farming household typologies in relation to participation in agri-environmental schemes, and outline the relationship between farming households and positive environmental action.

Full findings will be presented at the conference but are currently subject to information governance restrictions.

With an increasing interest in climate change and supporting the natural environment, our findings provide insight into the characteristics of farming households and their relationship to engagement with agri-environmental schemes. Our results will inform policy-makers seeking to support farming and the rural environment.

